# Autumn destabilization of deep porewater CO_2_ store in a northern peatland driven by turbulent diffusion

**DOI:** 10.1038/s41467-021-27059-0

**Published:** 2021-11-25

**Authors:** A. Campeau, D. Vachon, K. Bishop, M. B. Nilsson, M. B. Wallin

**Affiliations:** 1 Department of Forest Ecology and Management, Swedish University of Agricultural Sciences, Umeå, Sweden; 2 Department of Air, Water and Landscape, Uppsala University, Uppsala, Sweden; 3 Department of Ecology and Environmental Sciences, Umeå University, Umeå, Sweden; 4 Department of Aquatic Sciences and Assessment, Swedish University of Agricultural Sciences, Uppsala, Sweden

**Keywords:** Limnology, Carbon cycle, Hydrology

## Abstract

The deep porewater of northern peatlands stores large amounts of carbon dioxide (CO_2_). This store is viewed as a stable feature in the peatland CO_2_ cycle. Here, we report large and rapid fluctuations in deep porewater CO_2_ concentration recurring every autumn over four consecutive years in a boreal peatland. Estimates of the vertical diffusion of heat indicate that CO_2_ diffusion occurs at the turbulent rather than molecular rate. The weakening of porewater thermal stratification in autumn likely increases turbulent diffusion, thus fostering a rapid diffusion of deeper porewater CO_2_ towards the surface where net losses occur. This phenomenon periodically decreases the peat porewater CO_2_ store by between 29 and 90 g C m^−2^ throughout autumn, which is comparable to the peatland’s annual C-sink. Our results establish the need to consider the role of turbulent diffusion in regularly destabilizing the CO_2_ store in peat porewater.

## Abstract (web summary)

The CO_2_ stored in deep peat porewater is viewed as a fixed component of peatland C cycling. Here, the authors reveal a hitherto unknown hydro-physical process that results in sudden losses from this CO_2_ store every autumn.

## Introduction

Northern peatlands represent an important long-term global sink of atmospheric carbon dioxide (CO_2_) that has contributed to cool the Earth’s atmosphere throughout the Holocene^1–3^. This C sink arises from the primary production at the peatland surface, being higher than C mineralisation cumulated over the entire peat depth, which leads to C burial in the form of peat. Peatlands are typically viewed as two-layer systems^4^. They feature a highly active layer (acrotelm), which comprises the peatland’s surface (i.e. above the lowest water table), and an underlying layer (catotelm) of permanently waterlogged peat^5^. Most of the peatland CO_2_ cycling is considered to operate within the acrotelm^6^. There, living plants fix CO_2_ from the atmosphere and oxygen is freely available to fuel respiration that returns a fraction of this CO_2_ to the atmosphere. Water moves relatively rapidly through the partially decomposed peat in the acrotelm, hence, also generating most of the lateral CO_2_ export in runoff^7–10^. In comparison, the catotelm peat layer is generally cold and void of oxygen, which slows down decomposition^6^. The catotelm porewater is also constrained by, in general, low hydraulic conductivity with deeper, more decomposed and compacted peat^11^. Water in the catotelm porewater is typically rich in dissolved CO_2_, as a consequence of slow but relatively constant CO_2_ production coupled with even slower removal processes^12–14^. As long as CO_2_ remains confined in the catotelm, its role in the peatland CO_2_ cycling is negligible.

Transport of porewater CO_2_ from the catotelm to the acrotelm is mostly driven by diffusion^12,15,16^. This vertical diffusive transport is presumed to take place through molecular diffusion, a slow and constant process (diffusion coefficient *D* ~ 10^−9^ to 10^−8^ m^2^ s^−1^, depending on peat porosity and tortuosity^12,17^). Under strict vertical molecular diffusion, a molecule of CO_2_ generated a meter below the water table will take about 15 years to reach the acrotelm, where net losses by atmospheric CO_2_ emission or lateral hydrological CO_2_ export can take place. Hence, there is a general agreement that the catotelm peat porewater CO_2_ store contributes little to the seasonality in peatland CO_2_ cycling^18^. Studies documenting catotelm porewater CO_2_ dynamics in northern peatlands have hitherto relied solely on methodologies with a low temporal sampling resolution (e.g. refs. ^19–21^). The assumption of slow dynamics in catotelm porewater gas store has not been explicitly tested, which may result in overlooking key process controlling temporal dynamics in peatland CO_2_ cycling.

Here, we evaluate the stability of the catotelm porewater CO_2_ store in a boreal peatland using automated in situ sensor technology. Our data consist of hourly measurements over 4 consecutive years of porewater CO_2_ concentration and temperature at different depths in a 2-m-deep vertical peat profile. These data reveal rapid and regular losses of catotelm porewater CO_2_ recurring every autumn. We assess the rate of vertical diffusion, based on the heat budget method, and demonstrate that diffusion in the catotelm porewater occurs at orders of magnitude above the molecular rate of diffusion. Vertical diffusive transport occurs instead through turbulent diffusion, which reflects the presence of small-scale random fluid motion propagating through the peat porewater^22^. The regular weakening of porewater thermal stratification every autumn enhances turbulent diffusion, which results in rapid transport of deep porewater CO_2_ towards the surface. These findings reveal a hitherto unknown process that makes the catotelm and its porewater CO_2_ store far more dynamic than previously thought. The implications for the peatland CO_2_ cycle, however, remain to be fully investigated.

## Results and discussion

### Porewater CO_2_ and temperature dynamics

**Fig. 1:**
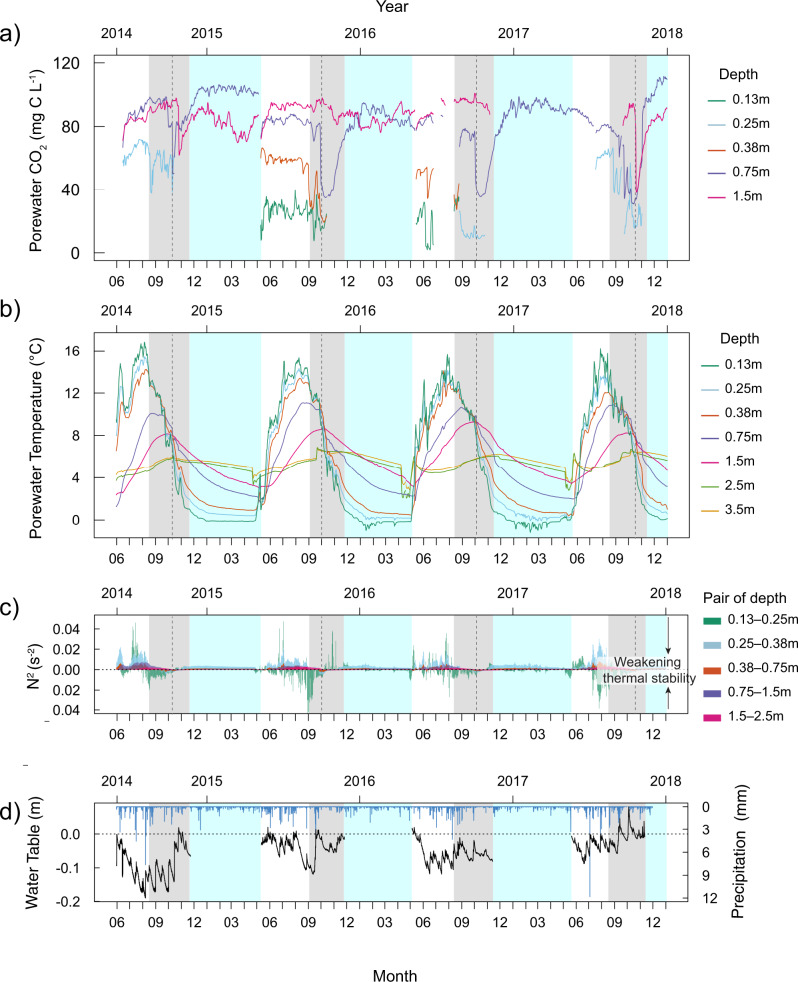
Porewater CO_2_ concentration, temperature, buoyancy frequency and hydrological conditions over time. Timeseries of the porewater **a** CO_2_ concentration (mg C L^−1^) and **b** temperature (°C) at each depth, **c** buoyancy frequency (N^2^, s^−2^) between pairs of adjacent porewater depths and **d** water table position (metres relative to ground surface) (black line) and hourly precipitation (mm) (blue lines), from June 2014 to January 2018. In **a** and **b**, each line represents a different depth relative to ground surface (0.13 m (green), 0.25 m (blue), 0.38 m (orange), 0.75 m (purple) and 1.5 m (magenta) below ground surface). In **b**, porewater temperature measurements at 2.5 m (light green) and 3.5 m (yellow) are also presented. In **c**, each coloured area represents a different pair of porewater depths 0.13 and 0.25 m (green area), 0.25–0.38 m (blue area), 0.38–0.75 m (orange area), 0.38–0.75 m (orange area), 0.75–1.5 m (purple area) and 0.75–1.5 m (purple area), 1.5–2.5 m (magenta area). The vertical dotted lines in (**a**–**c**), marks the day of weakest porewater thermal stability (i.e. equal temperatures from 0 to 1.5 m deep). In each panel, the x-axes indicate the dates, with years on top and month numbers on the bottom axis. The background areas, coloured in grey and cyan, mark the periods of weak thermal stability and ice/snow cover on the peatland surface, respectively.

**Fig. 2:**
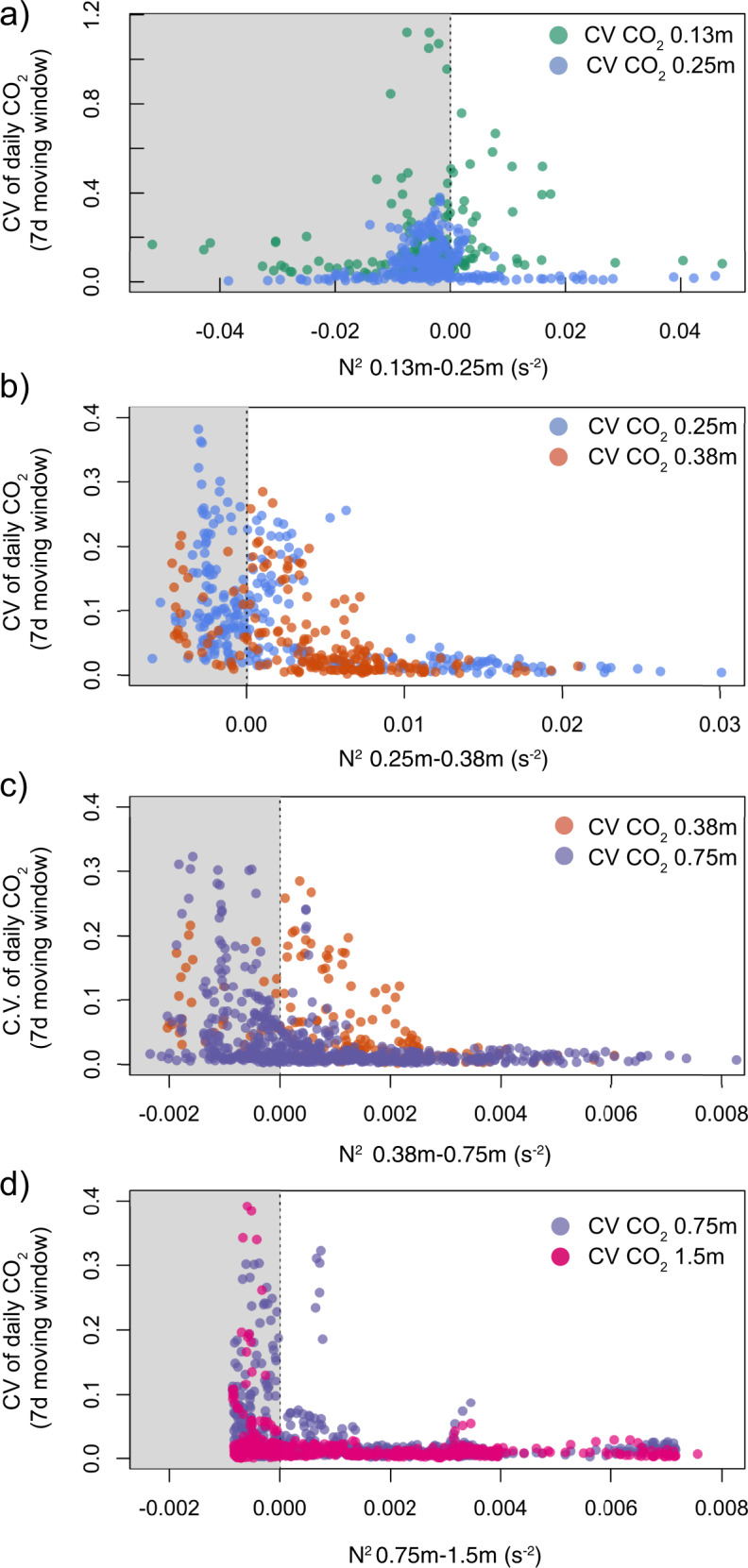
Relationship between porewater CO_2_ concentration variability and thermal stability. The 7-day moving coefficient of variation (C.V) for the daily averaged porewater CO_2_ concentration plotted against the daily averaged buoyancy frequency (N^2^) between different pairs of adjacent porewater depths: in **a** 0.13 and 0.25 m, **b** 0.25 and 0.38 m, **c** 0.38 and 0.75 m, **d** 0.75 and 1.5 m.

Porewater CO_2_ concentration timeseries reflect the constantly evolving balance between input and removal processes. The CO_2_ inputs in peat porewater occur through transport from adjacent layers and from local biogenic processes (e.g. rhizospheric and microbial production). The latter is mostly controlled by temperature, organic matter quality and oxygen or other electron acceptors availability^23^. Losses of porewater CO_2_ occurs mostly by transport processes that remove CO_2_ from the porewater (e.g. atmospheric emission and lateral hydrological export). Here we found that porewater CO_2_ concentration was lowest and most variable in the shallowest porewater (0.13 m in Fig. 1a, Fig. 2a), which is consistent with dynamic equilibrium between inputs and removal processes. Porewater CO_2_ concentration increased and became steadier with depth (Figs. 1a, 2), which is again consistent with slower and more constant input and removal processes. There were, however, events of large and sudden losses in porewater CO_2_ concentration recurring every autumn over the 4 consecutive years of observation that suggested a sudden rise in transport processes (Fig. 1a).

These events consisted of abrupt sequential decreases in peat porewater CO_2_ concentration along the peat depth profile. The large decreases in CO_2_ concentration first occurred in the surface porewater (0–0.25 m) between the end of August to September, and progressed down to deeper porewaters (0.75 m), around early October, and occasionally at 1.5 m depth later in November (Fig. 1a). The decreases in porewater CO_2_ concentration were most dramatic and regular at 0.75 m depth, only occurring near the end of September to mid-October (Fig. 1a). Outside of autumn, an isolated event of rapid decrease in CO_2_ concentration in the shallowest porewater (0.13–0.38 m depth) was identified between June 5th and 11th 2016. In early winter, the peat porewater CO_2_ concentration (only measured at 0.75 and 1.5 m depth) steadily returned to average growing season levels (Fig. 1a, Supplementary Fig. 1 and Supplementary Table 1) and remained relatively stable throughout winter and spring.

Periods of rapid decrease in porewater CO_2_ concentration always occurred together with the equilibration of porewater temperatures between two adjacent depths (Fig. 1a, b). The weakening of thermal stratification between water masses is associated with fluid instability. This instability can be expressed by the buoyancy frequency (N^2^, s^2^)^24^, whereby weak thermal stability between two porewater layers is indicated by N^2^ approaching zero (Fig. 1c). Periods where the N^2^ was near-zero coincided with periods with the most variable porewater CO_2_ concentrations (Fig. 2), hence reinforcing a link between the weakening of porewater thermal stability and losses from the CO_2_ store. Each event of rapid loss in porewater CO_2_, including those in autumn and in June 2016, corresponded with periods where N^2^ was near-zero (Fig. 2). The event in June 2016 occurred due to unusually cold weather conditions that equilibrated the surficial peat porewater temperatures for about 6 days (Fig. 1a–c).

Throughout much of the ice-free season, porewater temperatures were considerably warmer near the surface and decreased sharply with depth, thus generating a strong vertical thermal stratification (high thermal stability, high N^2^) (Fig. 1b, c). In autumn, the progressive equilibration of porewater temperature along the peat depth profile occurs due to surface cooling, which brings N^2^ near zero and weakens the thermal stability (Fig. 1b, c). The vertical equilibration of catotelm porewater temperature begins in the surficial porewater in mid-August [0.13–0.25 m] and progresses down the depth profile in October and November [0.75–1.5 m] (Fig. 1c), following the same sequence as the sudden losses of porewater CO_2_ concentration (Fig. 1a).

**Fig. 3:**
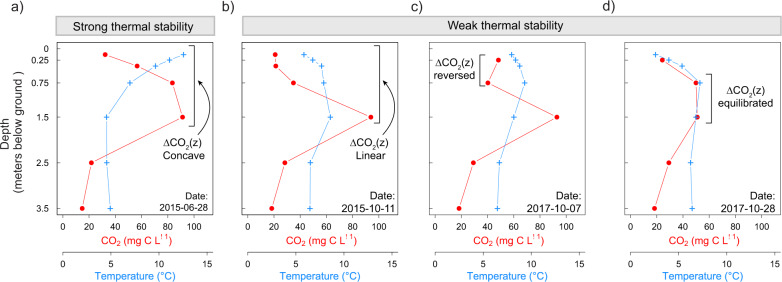
Shapes of the peat porewater CO_2_ concentration depth profile. Depth profile of the daily averaged porewater CO_2_ concentration (red circles and lines) and temperatures (blue crosses and lines) from 0 to 3.5 m below ground surface. Each panel presents a different example of depth profiles: **a** during strong thermal stability, **b** weak thermal stability, where the CO_2_ depth gradient (∆CO_2_(*z*)) is linear, **c** weak thermal stability, where the ∆CO_2_(*z*) between 0.25 and 0.75 m is reversed, and **d** weak thermal stability, where the ∆CO_2_(*z*) between 0.75 and 1.5 m is in equilibrium (i.e. almost null).

The large and rapid decreases in porewater CO_2_ concentration during periods of weak thermal stability dramatically altered the shape of the porewater CO_2_ depth profile. Throughout most of the growing season (high thermal stability, high N^2^), the porewater CO_2_ concentration increased sharply with depth, which resulted in a persistent convex profile from May to August (Fig. 3a). This convex vertical CO_2_ concentration gradient is consistent with slow vertical diffusive transport^17^. During the autumn, the porewater CO_2_ depth gradient suddenly became linear with depth (Fig. 3b) or sometimes collapsed completely between two adjacent porewater depths (Fig. 3d). There were also occasional inversions in the porewater CO_2_ depth gradient in autumn, during which CO_2_ concentration became higher in shallower porewater than in the deeper porewater (Fig. 3c). For example, the sudden decreases in porewater CO_2_ concentration at 0.75 m sometimes coincided with a brief increase in the surficial porewater CO_2_ (depths from 0.38 to 0.13 m) (Fig. 1a). The decrease in the CO_2_ depth gradient (going from convex to linear), together with the sequential fluctuations over the depth profile and occasional inversions strongly suggest a sudden increase in vertical diffusive transport of catotelm porewater CO_2_ towards the surface during periods of weakened thermal stability.

### A new consideration of turbulent diffusion

**Fig. 4:**
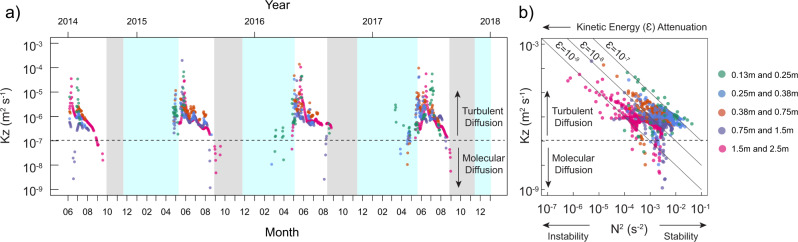
Vertical diffusion coefficient over time and as a function of thermal stability. **a** Timeseries of the apparent diffusion coefficient (*K*_app_; m^2^ s^−1^) between two adjacent porewater depths between June 2014 and January 2018, derived from the heat budget method and **b** scatterplot of the *K*_app_ in relation to the buoyancy frequency (N^2^; s^−2^) between the same pairs of porewater depths. Symbols are coloured based on the pairs of porewater depths, 0.13 to 0.25 m (green), 0.25 to 0.38 m (blue), 0.38 to 0.75 m (orange), 0.75 to 1.5 m (purple), 1.5 to 2.5 m (magenta). The horizontal dotted lines in both **a** and **b** mark the threshold between the turbulent and molecular rate of diffusion. Oblique full lines in **b** indicate the theoretical relationship between *K*_z_ and N^2^ described by Eq. (1)^24^, under different levels of kinetic energy input (*ε* = 10^−7^ to 10^−9^ m^2^ s^−3^) and a constant *γ*_mix_ of 0.10. In **a**, the x-axes indicate the dates, with years on top and month numbers on the bottom axis. In **a**, the background areas coloured in grey and cyan mark the periods of weak thermal stability and ice/snow cover on the peatland surface, respectively.

The direction of diffusive transport is dictated by concentration gradients (i.e. diffusing from areas of highest to lowest concentration). The speed at which this diffusion occurs, whether molecular or turbulent, depends on myriad environmental conditions. The molecular diffusion occurs at a low and constant speed (coefficient below 10^−7^ m^2^ s^−1 ^^12,15,17^), while the turbulent diffusion is faster and can varyorders of magnitude in both time and space (coefficient above 10^−7^ m^2^ s^−1^)^25^. Vertical diffusive transport of CO_2_ in the catotelm porewater is considered to take place mostly by molecular diffusion^12,15,16^. However, our estimates of the apparent diffusion coefficient (*K*_app_) in the peat porewater, based on the heat budget method^26^, occurs at orders of magnitude higher than is possible by molecular diffusion and varies widely over time (i.e. from 10^−7^ to 10^−4^ m^2^ s^−1^ (Fig. 4a)). Thus, we surmise that turbulent diffusion governs the vertical diffusive transport of catotelm porewater CO_2_. This implies that vertical CO_2_ diffusion is enhanced relative to the molecular rate by the presence of small-scale random fluid motion (i.e. turbulence) in the catotelm porewater.

According to Osborne^24^, the turbulent diffusion coefficient (*K*_z_) can be described as:

1 $${K}_{{{{{{\rm{z}}}}}}}={\gamma }_{{{{{{\rm{mix}}}}}}}\varepsilon /{{{{{{\rm{N}}}}}}}^{2}$$

where the turbulent diffusion coefficient (*K*_z_; m^2^ s^−1^) varies as a function of the kinetic energy dissipation rate (*ε*; m^2^ s^−3^) divided by the strength of the water density stratification (buoyancy (N^2^), s^−2^). In freshwater, buoyancy mostly reflects changes in vertical water temperature stratification. The mixing efficiency (*γ*_mix_) describes the fraction of energy storage as potential energy^24,27^. The kinetic energy production (here as equivalent to dissipation at steady state) provides a source of turbulence, but the strengthening and weakening of porewater thermal stability (N^2^) determine the degree of suppression or propagation of this turbulence (Eq. (1)^24^)^22^. We observed an increase in the *K*_app_ with decreasing N^2^, a relationship that is consistent with the model proposed by Osborne^24^ and previous observations from lakes^27,28^ (Eq. (1), Fig. 4b). The kinetic energy in the catotelm porewater was overall low (10^−7^ to 10^−9^ m^2^ s^−3^; Fig. 4b), which is about one order of magnitude lower than in small and sheltered northern lakes^29,30^. The kinetic energy was nonetheless higher in the near-surface porewater and attenuated with depth (Fig. 4b). This kinetic energy is likely supplied from wind shear near the surface and lateral water flow through the peat pores.

**Fig. 5:**
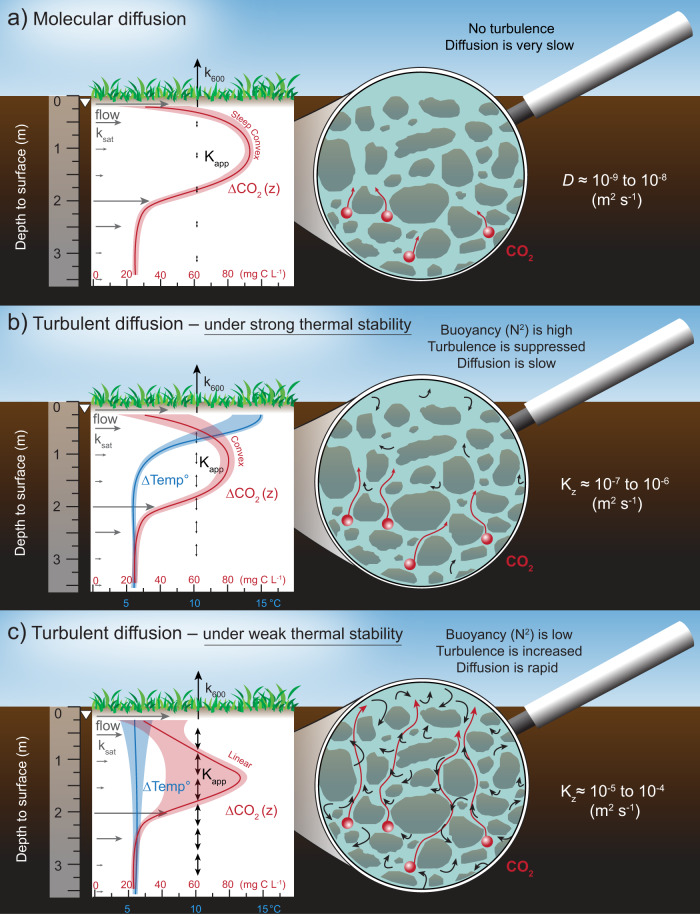
Schematic of the different rates of vertical CO_2_ diffusion in the catotelm porewater. From slowest to fastest: in **a** molecular diffusion, **b** turbulent diffusion during periods of strong porewater thermal stability (e.g. growing season) and **c** turbulent diffusion during periods of weak porewater thermal stability (e.g. autumn). Each panel shows the depth profile of porewater CO_2_ concentration (red line and area) and temperature (blue line and area), with the presumed saturated hydraulic conductivity (*k*_sat_, grey arrows) based on bulk density (Supplementary Fig. 2) and Campeau et al.^13^. Each panel also includes a magnifying glass illustrating the scale of vertical porewater CO_2_ diffusion (red spheres and arrows) and turbulence (black arrows) in the catotelm porewater. In **a**, the apparent diffusion coefficient (*K*_app_) is at the molecular rate, which is slow and constant over time (below 10^−7^ m^2^ s^−1^), resulting in a steep convex porewater CO_2_ depth gradient (∆CO_2_(*z*)). In **b**, turbulence is present in the porewater, but it is suppressed by the strong thermal stability (high buoyancy (N^2^)), which results in a slow vertical CO_2_ diffusion, nonetheless exceeding the molecular rate, yielding a persistently convex ∆CO_2_(*z*). In **c**, the weak thermal stability (low N^2^, *K*_app_ increases) allows turbulence to propagate in the catotelm, which enhances vertical porewater CO_2_ diffusion (upwards or downwards), potentially leading to variable ∆CO_2_(*z*).

Even under constant and low kinetic energy inputs, there was a three order of magnitude shift in the *K*_app_ with changing porewater thermal stability over time (N^2^), (Fig. 4b, Eq. (1)). The *K*_app_ ranged from 10^−7^ to 10^−6^ m^2^ s^−1^ during periods of strong thermal stability (May to August, high N^2^), to 10^−5^ to 10^−4^ m^2^ s^−1^ during periods of weak thermal stability (August to November, N^2^ near zero) (Figs. 4 and 5). The seasonality in turbulent diffusion implies that a molecule of CO_2_ generated 1 m below the water table can diffuse to the surface in just 1–2 h during periods of weak thermal stability (autumn), compared with 1.5 years during periods of strong thermal stability (growing season), and 15 years solely via molecular diffusion (Fig. 5). Combining the increase in turbulent diffusion in autumn with the steep vertical porewater CO_2_ concentration gradient that builds up over the growing season may lead to a rapid diffusion of catotelm porewater CO_2_ towards the surface where net losses occur.

Vertical equilibration of porewater temperature across the almost entire depth profile is unique to the autumn season and leads to a particularly weak porewater thermal stability (Fig. 1b). During most of the growing season, the surficial porewater is considerably warmer than the lower depths resulting in strong thermal stratification and stability. During winter and spring, an inverse vertical porewater temperature stratification is maintained by colder porewater near the ice and snow cover at the surface (Fig. 1b). Snow and ice cover throughout winter also shelters the porewater from kinetic energy input induced by wind or lateral flow, which contributes to suppressing turbulence in the peat porewater and increase stability in the porewater CO_2_ store. Hence, we suggest that unique conditions of weak porewater thermal stability in autumn lead to a sharp increase in turbulent diffusion that causes the recurring destabilizations and losses of the catotelm porewater CO_2_ store each autumn. A rapid diffusive transport of catotelm porewater CO_2_ towards the surface could enhance atmospheric CO_2_ emission and lateral hydrological CO_2_ export, causing the observed periodic loss of catotelm porewater CO_2_ store each autumn.

The recovery of porewater CO_2_ concentration in early winter was generally slow and steady (average rate of increase 0.05 (SD ± 0.12) and 0.04 (SD ± 0.07) mg C g^−1^ d^−1^ at 0.75 and 1.5 m, respectively, across all 4 years combined (Supplementary Table 1)). The return of porewater thermal stability and the onset of soil frost in winter decreases the vertical diffusion of porewater CO_2_ in early winter, which can lead to a recovery in concentration through local biogenicl production. The observed rate of recovery in porewater CO_2_ concentration during early winter is consistent with measurements of biogenic CO_2_ production in laboratory incubations at cold temperatures^31–33^. Similarly steady recoveries have been reported in northern lakes following ice-cover formation^34–36^. There were, however, days in 2014 and 2017 where the increase in the porewater CO_2_ concentration at 0.75 m was more sudden (rate between 0.2 and 1.2 mg C g^−1^ d^−1^) (Supplementary Fig. 1). The autumn of 2014 and 2017 were the only years where large and sudden losses in porewater CO_2_ occurred at 1.5 m, indicating that the destabilization of porewater CO_2_ store reached deeper depths in those two years. We therefore consider that those isolated days of more rapid recovery in porewater CO_2_ concentration at 0.75 m resulted from additional transport processes from subjacent or adjacent porewater and were not strictly explained by local biogenic production.

Turbulent diffusion has been studied extensively in lakes, where diffusion at the molecular level is almost always exceeded by turbulence in free-flowing water^27,28,37^. Turbulence is almost certainly on smaller scales in the catotelm porewater than in open water bodies (probably much lower than mm compared with mm to cm-scale eddies in lakes) (Fig. 5b, c). The increase in turbulent diffusion proposed in this study as a mechanism for the destabilization of porewater CO_2_ store in autumn should not be confused with the convective mixing that occurs in northern lakes in autumn (i.e. autumn lake turnover). While substantial losses of the dissolved CO_2_ store may occur in both lakes and peatlands during autumn, the hydrology of peatlands is critically different to that of a lake. In the case of lakes, the weakening of thermal stability in autumn also results in an increase in turbulent diffusion, but this increase is overridden by an increase in convection and advection^22^. The increased mass flow overturns the whole water column, causing large amounts of CO_2_ to be released to the atmosphere^28,36^. In peat porewater, mass flow is constrained by the low hydraulic conductivity and strong anisotropy of the peat. Hence, thermally driven convective mixing in peat porewater is only possible in areas where hydraulic conductivity is orders of magnitude higher than those measured across our studied peatland^38^ (Supplementary Fig. 3). Monthly measurements of the stable isotope ratio of porewater (δ^18^O) at this site confirms a persistent stratification of water masses across this depth profile in autumn (Supplementary Fig. 6). This lasting stratification does not indicate a simultaneous convective mixing with the increase in turbulent diffusion in autumn.

Some properties of the studied location could have made this peat depth profile prone to higher levels of turbulence in the catotelm porewater. The bulk density of the peat is slightly lower (0.016 g cm^−3^, Supplementary Fig. 2) than in most northern peatlands (range 0.02–0.25 g cm^−3 ^^39^) and other locations within the studied peatland (0.05 g cm^−3 ^^40^). The low bulk density of the peat is associated with a higher porosity (98%), which could potentially increase the propagation of turbulence in the catotelm. Our studied location is also found in a flow convergence zone and in proximity with the stream initiation point at the mire outlet, which may lead to high lateral water flow through the peat profile, supplying an additional source of kinetic energy production. Furthermore, a preferential flow path has been previously documented between 2 and 2.5 m depth^13^, suggesting potential further sources of kinetic energy production deep in the catotelm. However, these deep preferential flow paths, pipes and/or macropores are relatively common features in peatlands^41–43^. Thirdly, our studied peatland forms a large mire complex that is located at a topographic high point within the landscape, which might contribute to strong wind exposure (mean 2.6 m s^−1^, max 12.3 m s^−1^ over the full study period) that supplies additional kinetic energy and turbulence to the catotelm porewater.

### Other drivers of porewater CO_2_ dynamics

**Fig. 6:**
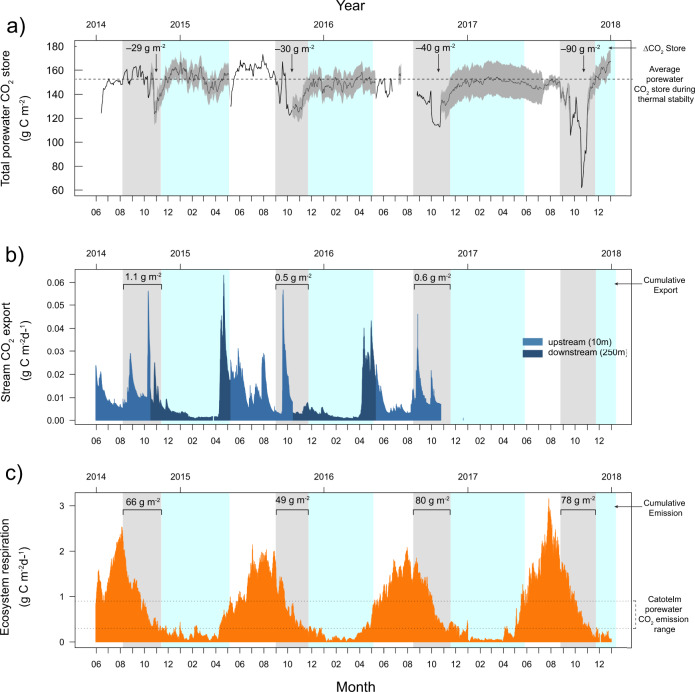
Catotelm porewater CO_2_ store compared with atmospheric and hydrological CO_2_ fluxes. Timeseries of the **a** estimated catotelm porewater CO_2_ store (g C m^−2^) per unit land area in the top 2 m of the peat profile, **b** stream CO_2_ export (g C m^−2^) per unit land area at the upstream (light blue) and downstream (dark blue) location relative to the stream initiation point, and **c** the ecosystem respiration (*R*_eco_, g C m^−2^) per unit land area based on eddy-covariance measurements from June 2014 to January 2018. In **a**, the grey area shows the possible range of porewater CO_2_ store for periods where measurements were unavailable in a certain layer while the black line shows the average porewater CO_2_ store. In each panel, the x-axes indicate the dates, with years on top and month numbers on the bottom axis. The background areas, coloured in grey and cyan, mark the periods of weak thermal stability and ice/snow cover on the peatland surface, respectively.

Other factors, such as changes in biological CO_2_ production, water table position, air-water gas exchange velocity and ebullition contribute to the temporal variability in porewater CO_2_ concentration. However, we consider those factors to have a relatively minor effect on the regular and rapid losses in catotelm porewater CO_2_ store each autumn compared with the increase in turbulent diffusion associated with weakening porewater thermal stability. Given the inhospitable conditions in the catotelm (e.g. energy substrate limitation and low amplitude in annual temperature), the apparent temperature response of peat decomposition is generally linear^44–46^ and sometimes inconsistent due to shifting metabolic pathways^33^. Annual temperature in the deep porewater vary across a narrow range (1–11 °C at 0.75 m and 2–9 °C at 1.5 m) with annual maxima being reached between August and October (Fig. 1b). It appears unlikely that the small and gradual changes in deep porewater temperature in autumn can cause the sudden losses in porewater CO_2_ observed in our data. Ecosystem respiration measurements at this site also indicate that substantial CO_2_ production still takes place across autumn despite the cooling temperatures (Fig. 6). Furthermore, the observed rate of loss in deep porewater CO_2_ store in autumn exceeds even the highest measurement of peat decomposition^23^, indicating that a complete shutdown of CO_2_ production cannot possibly explain the observed losses in catotelm porewater CO_2_ concentration. Together, this indicates that a possible decline in porewater CO_2_ production in autumn cannot fully explain the phenomenon present in our data.

Changes in water table position, which can be associated with CO_2_ dilution by rain water or changes in porewater flow direction, appear negligible in explaining the timing, depth, and magnitude of the changes in porewater CO_2_ store each autumn (Fig. 1d). Changes in water table position showed a rising, declining or stable water table at the time of the rapid drop in porewater CO_2_ concentration (Fig. 1d). The largest water table change represented a maximum 10% recharge of the total porewater volume contained in the top 2 m of the peat profile. In comparison, a refilling of 23 to 100% of the top 2 m of the peat porewater by CO_2_-poor rainwater would be needed to generate the observed drop in porewater CO_2_ store in autumn by dilution. While several studies have presented evidence of deep porewater recharge, with considerable influence on solutes concentration peat depth profiles^47–49^, the monthly measurements of porewater δ^18^O value at this site indicated only shallow rainwater infiltration (e.g., summer storm in August 2015 (^18^O enriched rain at 0.13 and 0.25 m depth); Supplementary Fig. 6). The ubiquitous radiocarbon enrichment of deep peat porewater dissolved organic carbon, CO_2_ and methane (CH_4_) relative to the surrounding solid peat is nonetheless clear evidence of dynamic solute transport at this site^13^ and in other peatlands^20,43,50^. Shifts in water table position could lead to changes in porewater flow direction, but such shifts typically occur over longer time-scales in response to persistent changes in water table position^51,52^. It therefore appears unlikely that flow direction suddenly reverses every autumn at this location. We nonetheless recommend high-resolution tracer studies to disentangle the effect of increased turbulent diffusion together with dilution and potential shifts in mass flow during periods of weak thermal stability.

There was a poor coherence between increases in wind speed and the timing of the losses in porewater CO_2_ store. Winds of high magnitude occurred across the whole year and were not limited to the periods of rapid losses in catotelm porewater CO_2_ store (Supplementary Fig. 5). While wind is most likely an important source of kinetic energy in the peat porewater, its effect on turbulence is greatly modulated by the thermal stability. Furthermore, estimates of the air-water gas exchange coefficient (*k*_600_) indicate low values, averaging 0.02 (SD ± 0.01) m d^−1^ (Supplementary Fig. 4), suggesting that air-water gas exchange mostly operates within a limited section of the surficial porewater. This estimate of *k*_600_ is on average one order of magnitude lower than those of small sheltered ponds, 0.19–0.72 m d^−1^, possibly its closest analogue^53^. Therefore, even an increase in air-water gas exchange due to high winds alone could not strip CO_2_ off the porewater at the depths observed in our data.

Lastly, ebullition is capable of rapidly mobilizing deep porewater gases directly to the atmosphere, a process that varies mostly with changes in atmospheric pressure and water table position^54–56^. Ebullition is most important for poorly soluble gases like CH_4_. In comparison, CO_2_ is about ten times more soluble than CH_4_, and thus mostly found in the dissolved rather than free-phase. Ebullition is therefore a comparatively weaker transport process for CO_2_ than CH_4_. However, it is also worth considering that ebullition in the catotelm could represent an additional source of kinetic energy and turbulence in the porewater, thus indirectly enhancing the vertical diffusion of porewater CO_2_ as a result. The various processes listed above certainly contribute to the variability in porewater CO_2_ concentration, but none could single-handedly explain the magnitude, regularity or depth of the changes in porewater CO_2_ store observed each autumn at this site.

### Implications for the peatland CO_2_ budget

The catotelm porewater CO_2_ store has been treated as a temporally stable feature of peatland C budgets^12,15,16^. In contrast, our results demonstrate that this porewater CO_2_ store (Fig. 6a) and the speed of vertical diffusion in the catotelm can vary widely across seasons (Fig. 4). The porewater CO_2_ store in the top 2 m of the catotelm dropped by between 29 and 90 g C m^−2^ during autumn when compared with the mean annual porewater CO_2_ storage during periods with strong thermal stability (152 ± 6 g C m^−2^) (Fig. 6a). These drops in catotelm porewater CO_2_ store are considerable compared with other flux components of the peatland CO_2_ budget, for example the net ecosystem exchange (NEE): –58 ± 21 g C m^−2^ yr^−1^ ^57^, and the annual stream CO_2_ export 3 ± 0.75 g C m^−2^ yr^−1^ ^58,59^. Losses in catotelm porewater CO_2_ store each autumn could therefore potentially contribute to the seasonality in peatland CO_2_ cycling.

We estimated that atmospheric emission from the catotelm porewater corresponded to an average flux of 0.3 g C m^−2^ d^−1^ across the year (i.e. based on the estimated average air-water gas exchange coefficient (*k*_600_; average 0.02 m d^−1^ (Supplementary Fig. 4)) and the average porewater CO_2_ concentration at 0.13 m depth (24 mg C L^−1^)). This average annual flux comprises 39% of the mean annual ecosystem respiration (0.67 ± 0.6 g C m^−2^ d^−1^), indicating that CO_2_ emissions from the catotelm porewater may control a significant share of the peatland-atmosphere CO_2_ exchange dynamics throughout the year. Allowing for the rapid diffusion of deeper catotelm porewater CO_2_ towards the surface, fostered by the sudden increase in turbulent diffusion in autumn, could increase this baseline flux by a factor of 3 (0.9 g C m^−2^ d^−1^) (i.e., assuming average *k*_600_ conditions and surficial porewater CO_2_ concentration corresponding to average concentrations observed at 0.75 m depth (84 mg C L^−1^)). Whether the weakening of thermal stability, increased wind shear, changing water table position or changing vegetation cover, could further enhance the *k*_600_ in autumn deserves closer examination. There was a steady decline in ecosystem respiration throughout autumn, but fluxes generally exceeded this range of catotelm porewater CO_2_ emission (Figure 6c). There were also multiple peaks in ecosystem respiration each autumn, which could have occurred in connection with the increase in turbulent diffusion during periods of weak thermal stability (Figure 6). The cumulative ecosystem respiration over the periods of weak thermal stability (49–80 g C m^−2^ d^−1^ across individual years (Fig. 6c)), was comparable to the periodic drops in porewater CO_2_ store each autumn (Fig. 6a). A more detailed investigation of the interplay between changes in porewater CO_2_ store and the peatland-atmospheric CO_2_ exchange is recommended to further elucidate these aspects.

Hydrological export is another key removal process of porewater CO_2_. The studied peat profile contains two layers of preferential lateral flow; the surficial peat and a deeper one located ~2 m below ground (*k*_sat_ in Fig. 5)^13,58^. Porewater at both of these depths contains less CO_2_ than the intermediate peat layers (0.5–1.5 m) (Fig. 1a). Rapid diffusion of porewater CO_2_ towards these two layers of preferential flow could increase CO_2_ export to the stream. Continuous measurements of stream CO_2_ concentration during the ice-free season recorded pulses of CO_2_−rich water into the stream during autumn (e.g., October 2014, August and September 2015, and September and October 2016 (Supplementary Fig. 7). However, the mass of CO_2_ exported to the stream outlet over the autumn appears negligible (0.5–1.1 g C m^−2^ yr^−1^) compared with the recurring losses from the porewater CO_2_ store during autumn (Fig. 6b). Periods of weak porewater thermal stability contribute nonetheless 25–53% of the long-term annual stream CO_2_ export (3 ± 0.7 g C m^−2^ yr^−1 ^^58^). Our studied peat profile is located in a zone of flow convergence and in close proximity to the stream initiation point (ca. 70-m distance), which could increase specific discharge at this site compared with other areas of the peatland. Areas of preferential flow are known for holding a disproportionate contribution to stream CO_2_ export relative to other areas of the peatland^41,60,61^. We therefore consider that hydrological export to the stream could still explain a small part of the observed losses in catotelm porewater CO_2_ store in autumn at this site. More detailed studies will be necessary to elucidate the full implications of changes in catotelm porewater CO_2_ store for the peatland C budget.

The high-frequency observations of porewater CO_2_ concentration provide evidence of regular destabilization of the catotelm porewater CO_2_ store during periods of weakened porewater thermal stability that recur every autumn. To date, molecular diffusion was considered the main vertical transport pathway for catotelm porewater CO_2_ towards the surface. Our analysis demonstrates that vertical diffusion of porewater CO_2_ in the catotelm occurs at rates orders of magnitude greater than is possible by molecular diffusion. Vertical CO_2_ diffusion occurs instead by turbulent diffusion, which is sensitive to changes in porewater thermal stability. This sensitivity can foster a sharp increase in porewater CO_2_ diffusion from the catotelm to the surface, hence causing a recurring loss of catotelm porewater CO_2_ store each autumn by atmospheric emission and hydrological export. We recommend further examination of the mechanism of turbulent diffusion in the catotelm of northern peatlands. Our findings reveal surprising dynamics in the catotelm porewater CO_2_ store in a northern peatland. The implication of such dynamism for the peatland C budget, in particular for methane emissions to the atmosphere, have yet to be fully resolved. The catotelm porewater CO_2_ store represents the equivalent of three consecutive years of net carbon accumulation by this peatland^59^. Thus, even slight fluctuations in the porewater CO_2_ store could have significant implications for the peatland CO_2_ sink capacity. The porewater of northern peatlands thus represents a dynamic, but possibly misunderstood component of peatland C cycling.

## Methods

### Study site

This study was conducted at Degerö Stormyr, a 6.5 km^2^ mire complex, located in Northern Sweden, at a topographic high point (~270 m.a.s.l.) about 60 km north-west of Umeå, Sweden (64°11′N, 19°33′E). The peatland is classified as an oligotrophic fen and is mostly undisturbed. Degerö Stormyr is composed of several inter-connected peatlands, separated by islets and ridges of glacial till soils. The study was conducted in a section draining 2.7 km^2^ of the total peatland complex, which is dominated by the fen (70%), but contains forested areas on the outskirts of the catchment (30%). Forested areas are about one km horizontal distance away from the studied peat depth profile. The peat depth profile consists of accumulated peat in the top 3 m, which overlays a ~1-m-thick layer of ancient organic lake sediment, for a total depth of about 4 m. The bulk density of the peat depth profile in the top 2 m is low and averages 0.016 ± 0.009 g cm^−3^, which corresponds to an average porosity of 98% (Supplementary Fig. 2). The peat depth profile is located ~70 m away from the initiation point of the stream outlet. The stream is found in an area of flow convergence where deep porewater is forced to the surface by a shallowing underlying mineral soil layer. The stream flow and C export are generated mostly through two conductive peat layers. The first consists of a preferential flow layer (i.e., deep macropore) at ~ 2 m below ground surface (Supplementary Fig. 2). The second is confined to the surface peat porewater [0–0.15 m below ground surface], with fluctuating contribution based on water table levels^13,58^.

Degerö Stormyr is part of the European research infrastructure Integrated Carbon Observation System (ICOS) and Swedish Infrastructure for Ecosystem Science (SITES), through which the site has acquired a long historical record of atmospheric CO_2_ and CH_4_ exchange via eddy-covariance based measurements (since 2001)^57,59,62^, hydrological C export^58^, and meteorological observations. The climate in this region is cold temperate humid, with a 30 year (1981–2010) mean annual precipitation of 614 mm and mean annual temperature of 1.8 °C. Maximum average temperature typically occurs in July (14.7 °C), while the minimum average temperature is usually reached in January (−9.5 °C)^63^. The peatland bears a persistent snow and ice cover from November to early May, with the ice typically reaching down to 10–30 cm below ground surface^64^. The underlying geology comprises base-poor Svecofennian metasediments/metagreywacke (Geological Survey of Sweden, Uppsala, Sweden). The vegetation field layer is dominated by lawn and carpet plant communities dominated by *Eriophorum vaginatum* L., *Trichophorum cespitosum* (L.) *Hartm*., *Vaccinium oxycoccos* L., *Andromeda polifolia* L., and *Rubus chamaemorus* L., The bottom layer consists ~100% of Sphagnum mosses, dominated by *Sphagnum balticum* (Russ.) C. Jens., *Sphagnum lindbergii* Schimp. in Lindb., *Sphagnum majus* (Russ.) C. Jens. and *Sphagnum papillosum* Lindb. in the lawn and carpets with *Sphagnum fuscum* (Schimp.) Klinggr. dominating the sparse hummocks and ridges.

### Peat depth profile instrumentation

A 4-m-deep peat profile was equipped with seven groundwater wells placed at different depths and screened for specific peat horizons via open slits along the wells ([0–0.25 m], [0.25–0.5 m], [0–0.5 m], [0.5–1 m], [1–2 m], [2–3 m], [3–4 m]). These wells were instrumented with CO_2_ sensors and thermistors. An additional tube, 1-m-deep, was installed and equipped with a pressure transducer (MJK 1400, 0–1 m, MJK Automation AB) for water level measurements. The top of the wells was sealed with thick rubber bungs. The seal was essential to prevent atmospheric gas exchange, but could potentially affect the water exchange within the tube when the groundwater table varied. However, we consider that the close fit between the slitted walls of the tube and the sensor allowed gases from the surrounding peat to diffuse into the tube making porewater gas measurements accurate. The wells had a 31 mm inner diameter, which allowed for a close fit (6.5 mm gap) around the CO_2_ sensors (18 mm diameter). The tubes were opened for sensor maintenance in May 2014 and 2015, to retrieve sensors in the shallow tubes in October of each year, and to redistribute sensors in August 2016 and September 2017.

Hourly measurements of the partial pressure of CO_2_ (*p*CO_2_) were made using the Vaisala CARBOCAP GMP221 nondispersive infrared (NDIR) CO_2_ sensors (range 0–20%). These sensors have been evaluated in soils and surface waters spanning a wide range in temperatures and ambient pressures^65^. The CO_2_ sensors were deployed at specific depths (0.13, 0.25, 0.38, 0.75, 1.5 m depth) in individual groundwater wells. Hourly CO_2_ measurements at 2.5 m depth were conducted, but only between June 14 and August 8, 2014. The deepest porewater (2–4 m below ground) was sampled manually for CO_2_ concentration on a monthly basis during the ice-free season in 2014 and 2015^13^. Sensor damage due to lightning strikes occasionally forced us to redistribute the sensors across the peat depth profile (e.g. measuring at 0.25 m instead of 0.13 and 0.38 m simultaneously). The CO_2_ sensors within the top 0.5 m of the peat profile were removed between November and May to prevent frost damage.

Each sensor was enclosed inside a water-tight, gas-permeable Teflon membrane (PTFE) and sealed with Plasti Dip (Plasti Dip international, Baine, MN, USA) to ensure that the sensor was protected from water, but remained permeable to gases. The Teflon membranes were replaced in May 2015 and 2016, following ice-melt. Concentrations of CO_2_ (expressed in mg C L^−1^) were determined from the *p*CO_2_ measurements considering water temperature (according to Henry’s law), hydraulic and atmospheric pressure^65^. Hourly porewater temperature measurements were conducted along the full 4-m depth profile (0.13, 0.25, 0.38, 0.75, 1.5, 2.5, 3.5 m depth) in individual wells using thermistors (TO3R, TOJO Skogsteknik). All continuously measured data were stored on an external data logger (CR1000, Campbell Sci.).

### Porewater CO_2_ store estimation

The total porewater CO_2_ store for the top 2 m of the peat depth profile was estimated by deriving the sum of the porewater CO_2_ store for three individual porewater layers (i.e. [0 to 0.5 m], [0.5 to 1 m], [1 to 2 m]). The porewater CO_2_ store for each porewater layer was calculated from the volume-weighted daily averaged CO_2_ concentration at different depths (i.e. (suming the average of 0.13 m and 0.38 m or 0.25 m for [0 to 0.5 m], 0.75 m for [0.5 to 1 m], 1.5 m for [1 to 2 m]). The CO_2_ store for each layer was adjusted for the porewater volume (average porosity 98%, Supplementary Fig. 2). For the surface porewater (0 and 0.5 m), the volume-weighting also included changes in water table position, in order to account for CO_2_ dilution and concentration with rising and falling water table. When CO_2_ concentration measurements were unavailable in a given layer (e.g. during winter between 0 to 0.5 m or periods following sensor damage), we applied the annual average CO_2_ concentration for these specific depths, together with the minimum and maximum, to derive a continuous range of possible porewater CO_2_ store.

### Thermal stability and turbulent diffusion

The thermal stability of the porewater was estimated using the buoyancy frequency (N^2^):

2 $${{{{{{\mathrm{N}}}}}}}^{2}=-\frac{g}{\rho }\frac{d\rho }{dz}$$

where *g* is the gravitational acceleration (m s^−1^), *ρ* is the density of porewater at a given depth (kg m^−3^), and *dρ* is the difference in density between the porewater at two different depths *dz*. The density of porewater was calculated based on water temperature assuming the absence of salinity. Periods of weak thermal stability were considered to begin when the difference in porewater temperature at 0.13 and 0.25 m was below 0.2 °C and ended when with freezing of the surface porewater (below 1 °C at 0.13 m). Periods of weak thermal stability therefore began in mid-August or early September, and ended in mid-November, lasting from 82 to 96 days across the 4 different years of observation. During periods of weak thermal stability, there were also weak inversions of the vertical porewater density gradient (i.e. slightly denser water above than below), which resulted in a negative vertical density gradient (*dρ*/*dz*) and N^2^. Under such conditions, it is possible for water in shallower peat to sink downwards because of gravity. However, we consider downward porewater flow (convection) in this peat profile to be small due to the sharp decrease of hydraulic conductivity of the peat with depth^38^ (Supplementary Fig. 3) and the limited length of time during which those inversions occurred. Monthly measurements of porewater stable isotope ratio did not indicate large-scale downward fluid motion during autumn (Supplementary Fig. 6).

Turbulent diffusion describes the vertical transport of mass, heat and momentum induced by random fluid motion (eddies), and is usually several orders of magnitude greater than molecular diffusion. An apparent diffusion coefficient (*K*_app_) can be derived using the vertically distributed peat porewater temperature time-series (i.e. heat budget method^26^). Vertical diffusive transport is considered to be turbulent when it exceeds the rate of molecular diffusion (i.e. >10^−7^ m^2^ s^−1^). In the absence of light, and assuming negligible lateral heat transfer, the vertical transfer of heat from one layer to the layer below is driven by diffusion. Vertical diffusion at depth *z* can be estimated as^27^:

3 $${K}_{{{{{{\mathrm{app}}}}}}}={\int }_{\max \,{{{{{\mathrm{depth}}}}}}}^{z}A(z^{\prime} )\frac{\partial T(z^{\prime} )}{\partial t}dz^{\prime} {\left[A(z)\frac{\partial T(z)}{\partial z}\right]}^{-1}$$

where *K*_app_ is the apparent diffusion coefficient (m^2^ s^−1^) and the temperature temporal gradient of the depths below $\frac{\partial T({z}^{{{\hbox{'}}}})}{\partial t}$ are calculated as the linear slope of temperature change over time, measured between seven days before and after the selected date (15 days in total), and $\frac{\partial T(z)}{\partial z}$ is the local (*z*) vertical daily average temperature gradient. *A* is the 2D planar area at depth. Daily *K*_app_ estimates were not considered when the vertical temperature gradient was no longer stratified, that is if the temperature slope has *r*^2^ < 0.7 or is not statistically significant (*p* > 0.05), and/or when the vertical temperature gradient is inverted (i.e. colder temperatures above warmer temperatures). The coefficient of variation (C.V.) of the porewater CO_2_ concentration was estimated over a 7-day moving window to assess the stability of porewater CO_2_ concentration at each porewater depth.

### Atmospheric and hydrological CO_2_ fluxes

We investigated the potential implications for the peatland C budget of a rapid diffusion of catotelm porewater CO_2_ towards the surface in autumn. We specifically quantified the poten tial emission of catotelm porewater CO_2_ to the atmosphere and the hydrological CO_2_ export to the stream outlet in the studied peatland. Atmospheric emission of dissolved porewater CO_2_ can be described using Fick’s first law, through which an estimate of the gas exchange coefficient (*k*_600_) at the air–water interface can be derived:

4 $${{k}}_{{{{{{{\mathrm{C}}}}}}}}{=}{{F}}_{{{{{{{\mathrm{C}}}}}}}}/({{C}}_{{{{{{{\mathrm{pw}}}}}}}}-{{C}}_{{{{{{{\mathrm{eq}}}}}}}})$$

Where *F*_C_ is the gas flux at the air–water interface, *k*_C_ is the gas exchange coefficient (see Eq. (5) below), *C*_pw_ is the gas concentration in the near-surface porewater and *C*_eq_ is the concentration of gas in porewater at equilibrium with the atmosphere. This equation was used to estimate the *k*_C_ between the surface porewater and the atmosphere. For *F*_C_, we used continuous measurements of atmospheric CH_4_ fluxes by eddy-covariance (ICOS; Degerö), since the peatland-atmosphere CH_4_ exchange is unidirectional (upward) and ascribed mostly to the anoxic water-saturated peat. In comparison, the atmospheric CO_2_ fluxes are bi-directional (both downward and upward) and largely influenced by primary production above ground making them unsuitable for estimating the *k*_C_. Steady ebulitive CH_4_ fluxes measured by the eddy-covariance flux tower, could lead to an overestimation of the *k*_C_, but this pathway of CH_4_ release is typically episodic^54,56^, thus mostly causing isolated overestimations. The range in *C*_pw_ was determined from the mean, maximum and minimum porewater *p*CH_4_ at 0.13 m depth^66^, while the *C*_eq_ was set at 1.7 ppm. Ambient *C*_pw_ and *C*_eq_ concentrations were determined according to porewater temperature (Henry’s law), hydraulic and atmospheric pressure.

The *k*_C_ was standardised for CO_2_ at 20 °C (*k*_600_) based on the following equation:

5 $${k}_{600}={k}_{{{{{{\mathrm{C}}}}}}}{(600/S{c}_{{{{{{{\mathrm{CH}}}}}}4}})}^{-2/3}$$

Where *Sc*_CH4_ is the Schmidt number based on the surface porewater temperature^67^. The eddy-covariance flux tower, where peatland-atmosphere CO_2_ and CH_4_ exchange is measured, is located on the same peatland but ~1 km away from the peat profile measurements, which could lead to important differences in C-exchange dynamics. However, both areas are characterized by a similar micro-topographical relief vegetation. The *k*_600_ estimate allowed us to determine an annual average rate of CO_2_ emission from the porewater to the atmosphere and estimate its possible increase due to the rise of turbulent diffusion in the catotelm porewater during autumn.

The hydrological CO_2_ export was estimated from the combined continuous stream CO_2_ concentration and discharge measurements, standardized by the estimated catchment area (2.7 km^2^). The stream CO_2_ concentration measurements were carried out using the same CO_2_ sensor methodology as used for the peat porewater described above. The sensors were deployed about 10 and 250 m downstream of the stream initiation point^13^. The stream CO_2_ concentration measurements in the upstream location were performed only during the ice-free season, but year round measurements were carried out in the downstream location. Stream CO_2_ export was estimated at both locations in order to derive a more complete estimate over time. The stream discharge was determined by applying a stage height-discharge rating curve to hourly water level measurements. Stream water height measurements were conducted throughout the year at a 10 m long trapezoidal flume inside a heated dam house ~50 m downstream of the stream initiation point. All calculations and analyses were performed using R (R Core Team, 2021)^68^.

## Supplementary information

Supplementary Information

Peer Review File

## Data Availability

The hourly measurements of porewater CO_2_ concentration, temperature and water table position, and estimates of daily average porewater CO_2_ concentration, coefficient of variation of porewater CO_2_ concentration, total porewater CO_2_ store and apparent diffusion coefficient, have been deposited in the Swedish National Data Service [10.5878/ggdt-ew12].

## Code availability

R codes related to this paper are available from the corresponding author upon request.

## References

[1] Frolking S, Roulet NT (2007). Holocene radiative forcing impact of northern peatland carbon accumulation and methane emissions. Glob. Change Biol..

[2] Ciais P (2011). Large inert carbon pool in the terrestrial biosphere during the Last Glacial Maximum. Nat. Geosci..

[3] Chapin FS (2000). Arctic and boreal ecosystems of western North America as components of the climate system. Glob. Change Biol..

[4] Morris PJ, Waddington JM, Benscoter BW, Turetsky MR (2011). Conceptual frameworks in peatland ecohydrology: looking beyond the two-layered (acrotelm-catotelm) model. Ecohydrology.

[5] Ingram HAP (1978). Soil layers in mires—function and terminology. J. Soil Sci..

[6] Blodau C (2002). Carbon cycling in peatlands—a review of processes and controls. Environ. Rev..

[7] Clymo RS (2004). Hydraulic conductivity of peat at Ellergower Moss, Scotland. Hydrological Process..

[8] Ingram HAP (1982). Size and shape in raised mire ecosystems: a geophysical model. Nature.

[9] Ivanov K. E. *Water Movement in Mirelands* (Academic Press Inc. (London) Ltd., 1981).

[10] Tipping E, Billett MF, Bryant CL, Buckingham S, Thacker SA (2010). Sources and ages of dissolved organic matter in peatland streams: evidence from chemistry mixture modelling and radiocarbon data. Biogeochemistry.

[11] Beckwith CW, Baird AJ, Heathwaite AL (2003). Anisotropy and depth-related heterogeneity of hydraulic conductivity in a bog peat. I: Laboratory measurements. Hydrological Process..

[12] Clymo RS, Bryant CL (2008). Diffusion and mass flow of dissolved carbon dioxide, methane, and dissolved organic carbon in a 7-m deep raised peat bog. Geochimica Et. Cosmochimica Acta.

[13] Campeau A (2017). Aquatic export of young dissolved and gaseous carbon from a pristine boreal fen: implications for peat carbon stock stability. Glob. Change Biol..

[14] Nilsson M, Bohlin E (1993). Methane and carbon dioxide concentrations in bogs and fens—with special reference to the effects of the botanical composition of the peat. J. Ecol..

[15] Ma S (2017). Data-constrained projections of methane fluxes in a northern Minnesota peatland in response to elevated CO_2_ and warming. J. Geophys. Res.: Biogeosciences.

[16] Walter BP, Heimann M (2000). A process-based, climate-sensitive model to derive methane emissions from natural wetlands: application to five wetland sites, sensitivity to model parameters, and climate. Glob. Biogeochemical Cycles.

[17] Clymo RS, Williams MMR (2012). Diffusion of gases dissolved in peat pore water. Mires Peat.

[18] Clymo RS, Pearce DME (1997). Methane and carbon dioxide production in, transport through, and efflux from a peatland. Philos. Trans. R. Soc. Lond. Ser. A: Phys. Eng. Sci..

[19] Blodau C (2007). Belowground carbon turnover in a temperate ombrotrophic bog. Glob. Biogeochem. Cycles.

[20] Wilson RM (2016). Stability of peatland carbon to rising temperatures. Nat. Commun..

[21] Steinmann P, Eilrich B, Leuenberger M, Burns SJ (2008). Stable carbon isotope composition and concentrations of CO_2_ and CH_4_ in the deep catotelm of a peat bog. Geochimica et. Cosmochimica Acta.

[22] Imboden, D. M. & Wüest, A. in *Physics and Chemistry of Lakes* (eds Lerman A. I. D. M. & Gat, J. R.) (Springer, 1995).

[23] Nilsson, M. & Öquist, M. in *Carbon Cycling in Northern Peatlands* (2013).

[24] Osborne TZ (1980). Estimates of the local rate of vertical diffusion from dissipation measurements. J. Phys. Oceanogr..

[25] Csanady, G. T. *Turbulent Diffusion in the Environment* (Springer Science & Business Media, 2012).

[26] Jassby A, Powell T (1975). Vertical patterns of eddy diffusion during stratification in Castle Lake, California1. Limnol. Oceanogr..

[27] Wuest A, Piepke G, Van Senden DC (2000). Turbulent kinetic energy balance as a tool for estimating vertical diffusivity in wind-forced stratified waters. Limnol. Oceanogr..

[28] Vachon, D., Langenegger, T., Donis, D. & McGinnis, D. F. Influence of water column stratification and mixing patterns on the fate of methane produced in deep sediments of a small eutrophic lake. *Limnol. Oceanogr.* **64**, 2114–2128 (2019).

[29] MacIntyre S (2021). Turbulence in a small boreal lake: consequences for air-water gas exchange. Limnol. Oceanogr..

[30] MacIntyre S, Crowe AT, Cortés A, Arneborg L (2018). Turbulence in a small arctic pond. Limnol. Oceanogr..

[31] Segura JH (2019). Microbial utilization of simple carbon substrates in boreal peat soils at low temperatures. Soil Biol. Biochem..

[32] Waddington, J. M., Rotenberg, P. A. & Warren, F. J. Peat CO_2_ production in a natural and cutover peatland: Implications for restoration. *Biogeochemistry* **54**, 115–130 (2001).

[33] Kolton M, Marks A, Wilson RM, Chanton JP, Kostka JE (2019). Impact of warming on greenhouse gas production and microbial diversity in anoxic peat from a Sphagnum-dominated bog (Grand Rapids, Minnesota, United States). Front Microbiol..

[34] Deshpande BN, Maps F, Matveev A, Vincent WF (2017). Oxygen depletion in subarctic peatland thaw lakes. Arct. Sci..

[35] Denfeld BA (2015). Temporal and spatial carbon dioxide concentration patterns in a small boreal lake in relation to ice cover dynamics. Boreal Environ. Res..

[36] Ducharme-Riel, V., Vachon, D., del Giorgio, P. & Prairie, Y. The relative contribution of winter under-ice and summer hypolimnetic CO_2_ accumulation to the annual CO_2_ emissions from northern lakes. *Ecosystems* **18**, 547–559 (2015).

[37] MacIntyre S, Flynn KM, Jellison R, Romero JR (1999). Boundary mixing and nutrient fluxes in Mono Lake, California. Limnol. Oceanogr..

[38] Rappoldt C (2003). Buoyancy-driven flow in a peat moss layer as a mechanism for solute transport. Proc. Natl Acad. Sci. USA.

[39] Rezanezhad F (2016). Structure of peat soils and implications for water storage, flow and solute transport: a review update for geochemists. Chem. Geol..

[40] Larsson, A., Segerstro, M. U., Laudon, H. & Nilsson, M. B. Holocene carbon and nitrogen accumulation rates in a boreal oligotrophic fen. *Holocene* **27**, 811–821 (2016).

[41] Holden J, Burt TP (2002). Piping and pipeflow in a deep peat catchment. Catena.

[42] Chason DB, Siegel DI (1986). Hydraulic conductivity and related physical-properties of peat, Lost River Peatland, northern Minnesota. Soil Sci..

[43] Glaser PH (2016). Climatic drivers for multidecadal shifts in solute transport and methane production zones within a large peat basin. Glob. Biogeochem. Cycles.

[44] Wu, Q., Ye, R., Bridgham, S. D. & Jin, Q. Limitations of the Q10 coefficient for quantifying temperature sensitivity of anaerobic organic matter decomposition:a modeling based assessment. *J. Geophys. Res. Biogeosci.* **126**, e2021JG006264 (2021).

[45] Scanlon D, Moore T (2000). Carbon dioxide production from peatland soil profiles: the influence of temperature, oxic/anoxic conditions and substrate. Soil Sci..

[46] Bergman I, Lundberg P, Nilsson M (1999). Microbial carbon mineralisation in an acid surface peat: effects of environmental factors in laboratory incubations. Soil Biol. Biochem..

[47] Griffiths NA, Sebestyen SD (2016). Dynamic vertical profiles of peat porewater chemistry in a northern Peatland. Wetlands.

[48] Peralta-Tapia A, Sponseller RA, Tetzlaff D, Soulsby C, Laudon H (2015). Connecting precipitation inputs and soil flow pathways to stream water in contrasting boreal catchments. Hydrological Process..

[49] Levy ZF, Siegel DI, Dasgupta SS, Glaser PH, Welker JM (2014). Stable isotopes of water show deep seasonal recharge in northern bogs and fens. Hydrological Process..

[50] Chanton JP (1995). Radiocarbon evidence for the substrates supporting methane formation within northern Minnesota peatlands. Geochimica et. Cosmochimica Acta.

[51] Siegel DI, Reeve AS, Glaser PH, Romanowicz EA (1995). Climate-driven flushing of pore water in peatlands. Nature.

[52] Fraser CJD, Roulet NT, Lafleur M (2001). Groundwater flow patterns in a large peatland. J. Hydrol..

[53] Holgerson MA, Farr ER, Raymond PA (2017). Gas transfer velocities in small forested ponds. J. Geophys. Res.: Biogeosciences.

[54] FechnerLevy EJ, Hemond HF (1996). Trapped methane volume and potential effects on methane ebullition in a northern peatland. Limnol. Oceanogr..

[55] Glaser PH (2004). Surface deformations as indicators of deep ebullition fluxes in a large northern peatland. Glob. Biogeochem. Cycles.

[56] Tokida T (2007). Falling atmospheric pressure as a trigger for methane ebullition from peatland. Glob. Biogeochem. Cycles.

[57] Peichl M (2014). A 12-year record reveals pre-growing season temperature and water table level threshold effects on the net carbon dioxide exchange in a boreal fen. Environ. Res. Lett..

[58] Leach JA, Larsson A, Wallin MB, Nilsson MB, Laudon H (2016). Twelve year interannual and seasonal variability of stream carbon export from a boreal peatland catchment. J. Geophys. Res.: Biogeosciences.

[59] Nilsson M (2008). Contemporary carbon accumulation in a boreal oligotrophic minerogenic mire—a significant sink after accounting for all C-fluxes. Glob. Change Biol..

[60] Holden J (2012). Natural pipes in blanket peatlands: major point sources for the release of carbon to the aquatic system. Glob. Change Biol..

[61] Dinsmore KJ (2011). Greenhouse gas losses from peatland pipes: a major pathway for loss to the atmosphere?. J. Geophys. Res.: Biogeosciences.

[62] Sagerfors J (2008). Annual CO_2_ exchange between a nutrient-poor, minerotrophic, boreal mire and the atmosphere. J. Geophys. Res.: Biogeosciences.

[63] Laudon H (2013). The Krycklan Catchment Study—a flagship infrastructure for hydrology, biogeochemistry, and climate research in the boreal landscape. Water Resour. Res..

[64] Granberg G, Ottosson-Löfvenius M, Grip H, Sundh I, Nilsson M (2001). Effect of climatic variability from 1980 to 1997 on simulated methane emission from a boreal mixed mire in northern Sweden. Glob. Biogeochem. Cycles.

[65] Johnson MS (2010). Direct and continuous measurement of dissolved carbon dioxide in freshwater aquatic systems—method and applications. Ecohydrology.

[66] Campeau A (2018). Stable carbon isotopes reveal soil-stream DIC linkages in contrasting headwater catchments. J. Geophys. Res.: Biogeosciences.

[67] Wanninkhof R (1992). Relationship between wind speed and gas exchange over the ocean. J. Geophys Res..

[68] R Core Team. R: A language and environment for statistical computing. R Foundation for Statistical Computing, Vienna, Austria, https://www.R-project.org/ (2021).

