# Supplementary material for: Autumn destabilization of deep porewater CO2 store in a northern peatland driven by turbulent diffusion
Source: Nat Commun. 2021 Nov 25;12:6857. doi: 10.1038/s41467-021-27059-0 (PMC8616934; doi:10.1038/s41467-021-27059-0)
Supplement: Supplementary file 1 — Supplementary Information [file 41467_2021_27059_MOESM1_ESM.pdf]

Supplementary Information:

Autumn destabilization of deep porewater CO_2 store in a northern peatland driven by turbulent diffusion

A. Campeau [1,2]*, D. Vachon [3], K. Bishop [4], M.B. Nilsson [1] and M.B. Wallin [2,4]

[1] Department of Forest Ecology and Management, Swedish University of Agricultural Sciences, Umeå, Sweden.

[2.] Department of Air, Water and Landscape, Uppsala University, Sweden.

[3] Department of Ecology and Environmental Sciences, Umeå University, Umeå, Sweden

[4] Department of Aquatic Sciences and Assessment, Swedish University of Agricultural Sciences, Uppsala, Sweden.

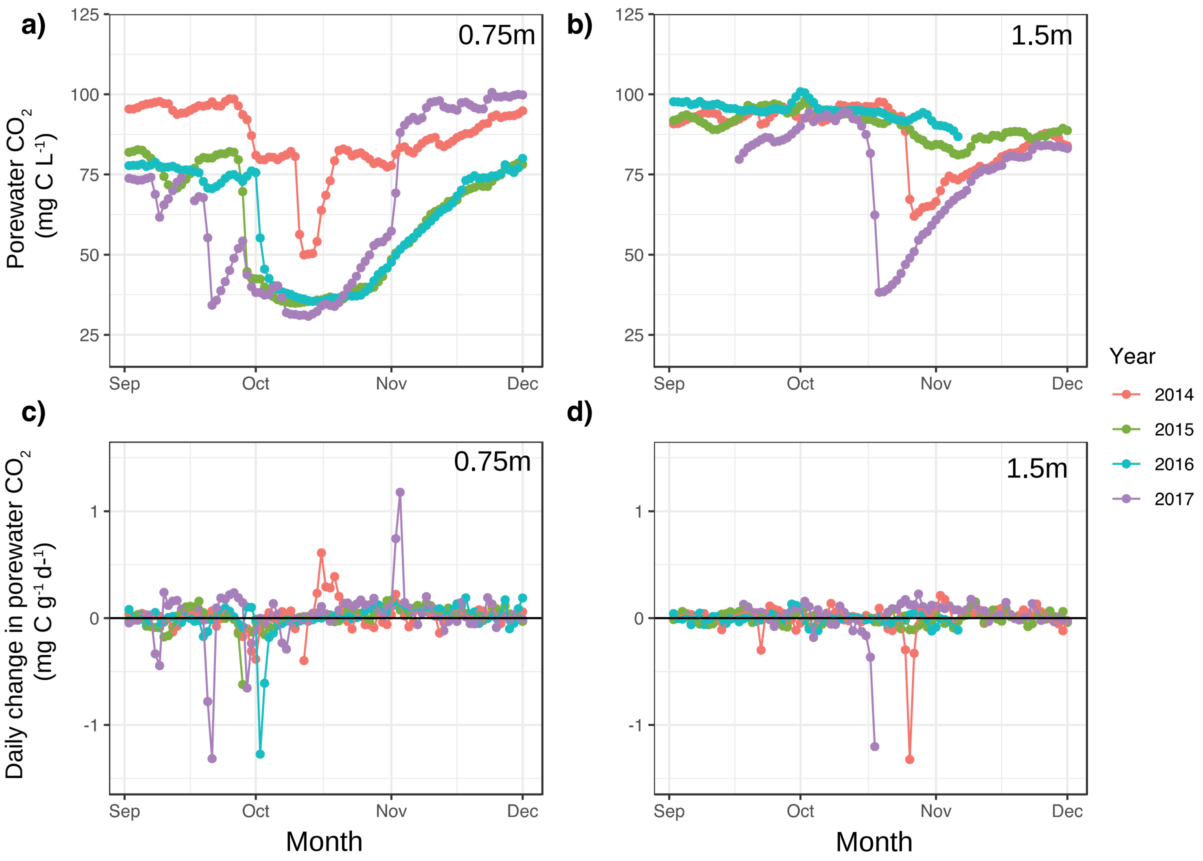

Supplementary Figure 1: Timeseries of the a,b) porewater CO_2 concentration (mg C L-1) and c, d) corresponding daily changes (mg C g^{-1} d^{-1}) at 0.75m (a,c) and 1.5m deep (b,d) between September and December on different years. Individual timeseries are coloured based on their years (2014 (red), 2015 (green), 2016 (blue), 2017 (purple)).

Supplementary Table 1: Rate of recovery over the 60 days following the rapid loss in porewater CO_2 concentration in autumn at 0.75m and 1.5m deep expressed in mg C g^{-1} d^{-1}, presenting both the average and standard deviation (±) on individual years (2014 to 2017). Data are presented in detail in Figure S1. Rapid losses in porewater CO_2 at 1.5m deep did not occur in 2015 and 2016.

CO_2 Recovery in Winter	Year	2014		2015		2016		2017	
	Unit	mg C g^{-1} d^{-1}		mg C g^{-1} d^{-1}		mg C g^{-1} d^{-1}		mg C g^{-1} d^{-1}	
	Parameter	Average	(±SD)	Average	(±SD)	Average	(±SD)	Average	(±SD)
Depth	0.75m	0.05	(±0.12)	0.04	(±0.06)	0.05	(±0.07)	0.07	(±0.19)
Depth	1.5m	0.03	(±0.06)	–		–		0.05	(±0.07)

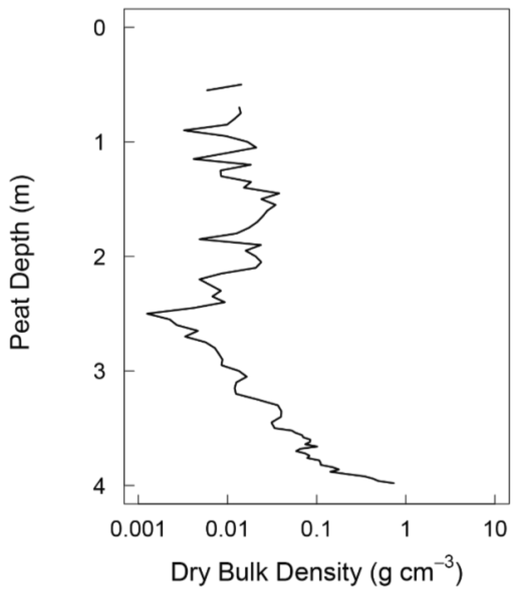

Supplementary Figure 2. dry bulk density (g cm^{-3}) of the peat depth profile at the studied location from the ground surface (0 m) to 4 m below ground where the bedrock is reached.

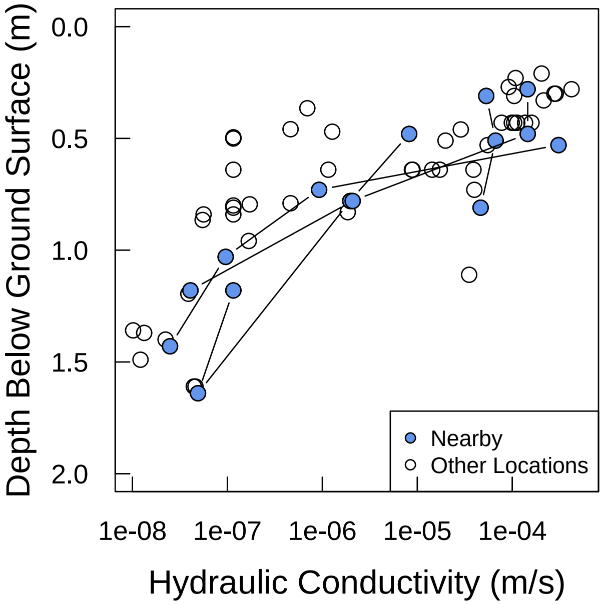

Supplementary Figure 3: Depth profile of the measured horizontal hydraulic conductivity inferred based on *methodology from [Nijp et al., 2019]* using piezometer water table depth measurement. Measurements collected in a nearby section of the peatland are marked with the full blue circles while measurements carried out at other locations are shown in open circles.

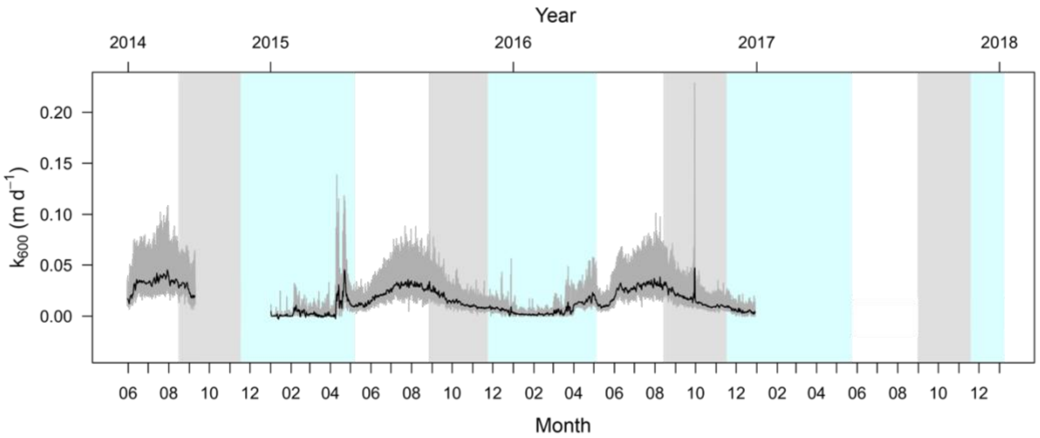

Supplementary Figure 4: Estimated average and range in air-water gas exchange coefficient (k_{600}) (black line and grey area, respectively). the x-axes indicate the dates, with years on top and month numbers on the bottom axis. The background areas, coloured in grey and cyan, marks the periods of weak thermal stability and ice/snow cover on the peatland surface, respectively

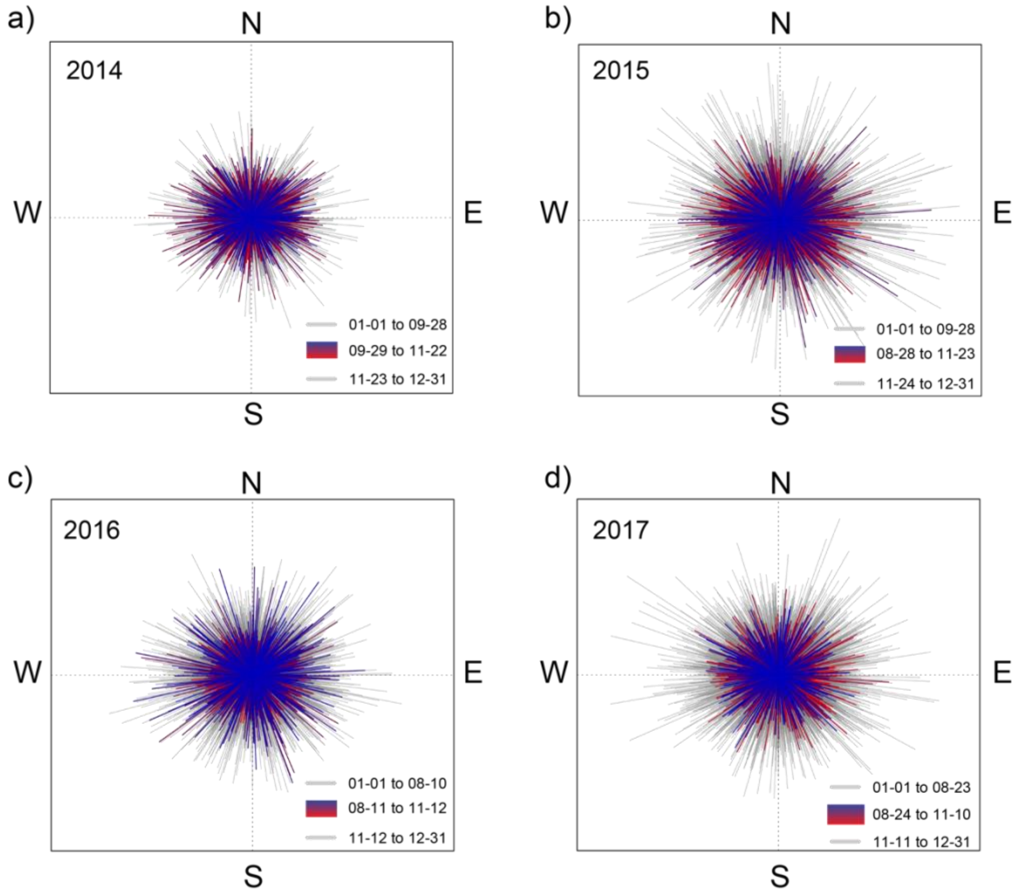

Supplementary Figure 5: Map of hourly wind vector in a) 2014, b) 2015, c) 2016 and d) 2017 measured by eddy-covariance (ICOS, Degerö). Grey lines show measurements during the periods of strong porewater thermal stability (growing season and winter), while periods of weak thermal stability are identified in blue-red shades.

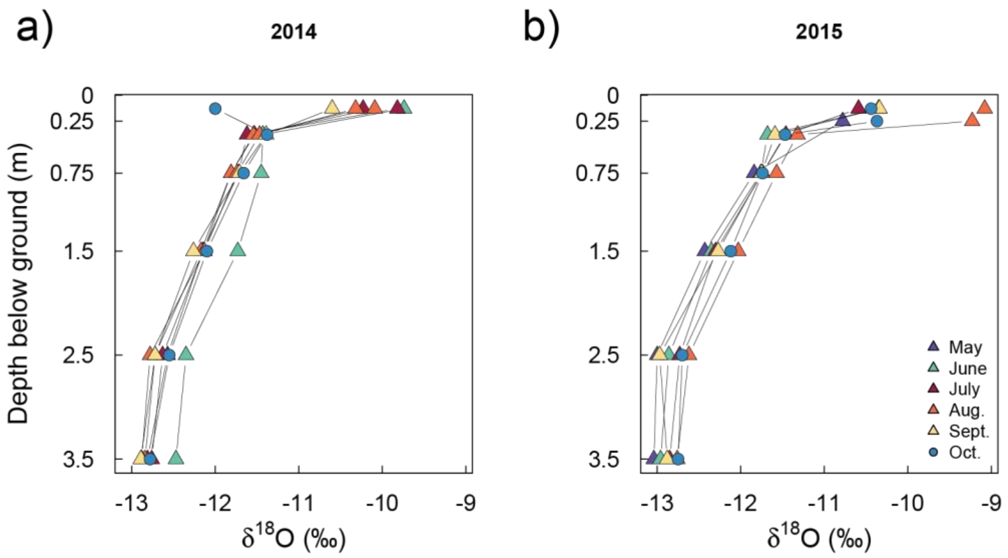

Supplementary Figure 6: Porewater stable isotope ratio ($\delta^{18}O$ ‰) measurement at different porewater depths (0 to 3.5m deep) in 2014 (a) and (b) 2015. Symbols are colored according to the month of sampling. Samples collected in October are marked with blue circles.

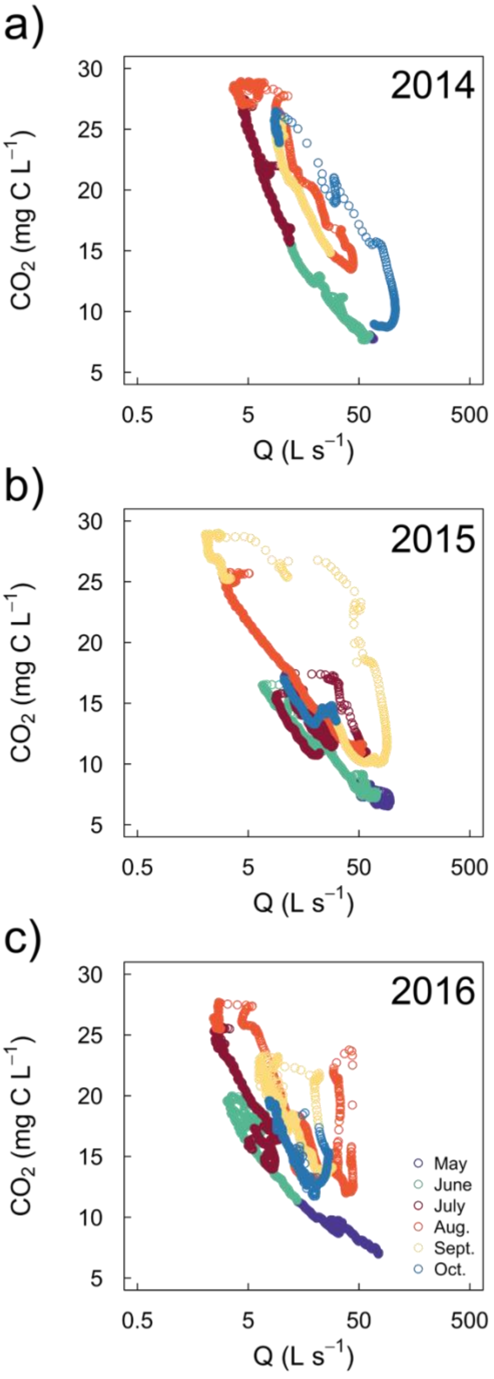

Supplementary Figure 7: Scatterplots of hourly measurements of stream water CO_2 concentration against stream discharge (L s^{-1}) during the ice-free season (May to October) at the upstream location (10m downstream from stream initiation point) in a) 2014, b) 2015 and c) 2016. Symbols are colored based on the month of the measurements, following the same code at in Figure S5.

Supplementary References

Campeau, A., K. Bishop, M. B. Nilsson, L. Klemedtsson, H. Laudon, F. I. Leith, M. Öquist, and M. B. Wallin (2018), Stable Carbon Isotopes Reveal Soil-Stream DIC Linkages in Contrasting Headwater Catchments, *Journal of Geophysical Research: Biogeosciences*, *123*(1), 149-167.

Nijp, J. J., K. Metselaar, J. Limpens, H. M. Bartholomeus, M. B. Nilsson, F. Berendse, and S. E. A. T. M. Zee (2019), High-resolution peat volume change in a northern peatland: Spatial variability, main drivers, and impact on ecohydrology, *Ecohydrology*, *12*(6).
